# Supplementary material for: Longitudinal Study to Assess the Quantitative Use of Fundus Autofluorescence for Monitoring Disease Progression in Choroideremia
Source: J Clin Med. 2021 Jan 11;10(2):232. doi: 10.3390/jcm10020232 (PMC7826764; doi:10.3390/jcm10020232)
Supplement: Supplementary file 1 [file jcm-10-00232-s001.pdf]

Supplementary Table 1

	Latest visual acuity		Baseline	Follow-up age	Follow-up interval	Baseline FAF area (mm^2)		Follow-up FAF area (mm^2)		RAE (mm^2/y)		
	OD	OS	age (y)	(y)	(months)	OD	OS	OD	OS	OD	OS	Mutation status and type
1	0.18	0.18	38	41	30	4.27	6.76	2.91	5.07	0.54	0.67	c.529delG p.E1777KfsX Deletion
2	2.7	1.78	48	50	31	0.2	0.33	0.1	0.17	0.04	0.06	Deletion of exon 10-11
3*	0.48	0.3	47	49	22	1.13	1.6	1.01	1.6	0.07	0.00	Deletion of exon 13-15.
4	0.18	0.6	50	55	54	2.67	2.53	2.03	1.62	0.14	0.20	c.535_538delGAAA p.E179Tfs Deletion
5b	2.3	0.78	29	32	38	NA	0.81	NA	0.67	NA	0.04	Deletion of exon 1
6	-0.08	-0.08	33	42	104	17.6	9.58	9.85	4.8	0.89	0.55	c.649_652delTACT p.Y217HfsX Deletion
7	2.3	1.3	52	54	27	4.56	5.08	3.76	4.79	0.35	0.13	c.649_652delTACT p.Y217HfsX Deletion
8	0.48	0.18	26	30	41	17.3	15.31	12.2	10.93	1.48	1.27	REP1 deletion
9*	0	0	23	26	36	16.08	11.81	11.97	8.15	1.38	1.23	c.1245_1246delins14 p.K415NfsX Deletion
10	0.18	0	17	19	25	20.26	21.76	15.73	16.85	2.21	2.40	Deletion of exons 1-11
11	0.78	0.3	56	58	25	11.18	13.61	8.98	9.62	1.07	1.93	c.1584_1587delTGTT p.P529Hfs Deletion
12	0.6	0.3	60	68	88	14.84	26.21	6.94	12.05	1.08	1.93	Deletion of exon 10-15
13ab	0.1	0	18	20	25	54.01	48.12	42.69	41.15	5.43	3.35	REP1 deletion
14	0.18	0.18	54	55	12	22.42	43.21	20.96	39.06	1.44	4.15	c.737_741delTAATC p.L246Qfs Deletion
15	0.18	0.18	23	27	48	12.24	11.09	8.27	7.09	0.99	1.00	Deletion of exon 12-13
16b	0.18	0.18	62	66	49	8.26	7.76	6.21	5.18	0.50	0.63	c.652_655delTCAC p.S218Kfs Deletion
17	0.1	0.2	19	20	7	59.89	53.74	54.37	48.18	9.46	9.53	c.1079delA p.N360TfsX Deletion
18	0	0.48	49	52	36	27.28	20.14	21.96	13.91	1.77	2.08	Deletion of exon 3-15
19b	0.3	0.18	20	21	12	8.44	9.11	7.31	7.7	1.14	1.42	Deletion of exon 5-8
20*	0.2	0.2	16	18	17	18.55	17.45	15.03	14.4	2.49	2.16	Deletion of exon 3
21*	0.14	0.16	10	11	6	30.68	27.24	27.3	19.74	6.76	15.00	Deletion of exon 2-7
22	0.3	1.9	47	49	28	0.41	0.57	0.31	0.48	0.04	0.04	c.1300_1303delGTGG p.V434Rfs Deletion
23	1	0.3	64	67	29	0.19	0.09	0.16	0.05	0.01	0.02	c.525_526delAG p.E177Kfs Deletion
24	0.6	1	51	52	18	2.29	2.05	1.58	1.51	0.47	0.36	c.1079delA p.N360Tfs Deletion
25	0.3	0.3	29	32	34	7.04	7.91	4.72	7.05	0.83	0.31	Deletion exon 9
26	0.6	0	20	22	15	1.29	2.34	1.11	1.98	0.14	0.29	c.525_526delAG p.E177Kfs Deletion
27	0.18	0.48	28	30	30	7.91	NA	6.33	5.07	0.64	NA	Deletion exon 3-4
28	0	0	19	22	33	27.95	27.37	24.49	21.88	1.27	2.02	c.492_493delGA p.N165Cfs Deletion

29	0	0.78	17	18	14	22.5	22.38	20.88	21.54	1.37	0.71	c.492_493delGA p.N165Cfs Deletion
30	0.18	0.18	31	34	34	1.26	0.84	0.92	0.53	0.12	0.11	Deletion exon 9
31	0	0	42	44	24	26.57	30.1	26.4	28.49	0.09	0.81	c.1762_1765delGTCA p.V588Nfs Deletion
32	0	1.48	37	41	49	3.51	0.67	2.78	0.47	0.18	0.05	Deletion of exon 10-15
33	0	0	21	22	6	18.4	27.59	17.54	25.96	1.67	3.26	c.886delA p.M296Kfs Deletion
34	0	0	34	37	31	9.91	9.9	9.53	NA	0.15	NA	c492_493delGA p.N165Cfs Deletion
35	0	0.3	28	29	10	15.1	16.34	13.51	14.23	1.89	2.51	Deletion of exon 1-15
36b	0	0.18	49	50	18	3.22	4.1	2.47	3.49	0.50	0.41	c.872_880del p.E291Vfs, Deletion
37	0.1	0.48	53	54	12	3.37	2.12	2.51	1.67	0.83	0.44	c.652_655delTCAC p.S218Kfs Deletion
38	0.1	0.3	32	34	13	17.82	20.87	17.35	16.09	0.42	4.31	Deletion of exon 1-15
39	-0.08	-0.08	21	22	12	76.96	78.09	53.29	52.14	22.92	25.13	c.179delA p.K60Rfs Deletion
40a	0.78	0.6	56	58	29	8.68	19.72	7.19	18.11	0.62	0.67	REP1 deletion
41a	0	-0.08	59	60	6	6.38	12.08	6.06	11.79	0.64	0.58	REP1 deletion
42a	0.6	0	20	22	15	1.29	2.34	1.11	1.98	0.14	0.29	REP1 deletion
43	0.48	0.48	56	57	9	3.24	10.25	3.12	9.85	0.16	0.53	c.1335dupA p.R446Tfs, Duplication
44*	2.7	0.78	47	52	63	NA	1.43	NA	0.41	NA	0.19	c.495dupT p.N165Cfs, Duplication
45	NA	NA	41	42	10	9.33	14.1	8.25	13.68	1.34	0.52	Duplication of exons 1-2 and 9-12
46	0	0	12	14	25	29.69	35.52	24.01	24.34	2.73	5.37	c.931GinsA, p.Y312Ifs, Insertion
47	0.1	0	25	26	9	17.14	13.3	15.95	12.66	1.56	0.84	c.1099_1100insTACC p.R367Lfs Insertion
48	NA	NA	68	70	12	4.38	0.41	3.66	0.34	0.70	0.07	c.1520A>G p.H507R Missense
49	0.6	1	42	46	39	0.6	0.41	0.5	0.33	0.03	0.02	c.715C>T p.R239X Nonsense
50	0.18	0.18	52	58	74	12.98	13.06	10.18	10.05	0.46	0.49	c.715C>T p.R239X, Nonsense
51	0	-0.08	22	24	26	3.67	5	2.72	3.52	0.44	0.68	c.126C>G p.Tyr42X, Nonsense
52	-0.08	-0.08	35	38	41	10.14	14.31	7.89	11.66	0.66	0.78	c.1264C>T p.Q422X, Nonsense
53	0.18	-0.08	37	40	24	23.29	37.31	19.98	31.98	1.63	2.63	c.877C>T p.R293X, Nonsense
54	2.3	2.7	48	51	34	1.56	1.46	0.95	1.07	0.21	0.14	c.877C>T p.R293X, Nonsense
55	0.78	0.3	56	60	47	41.48	20.43	31.47	15.33	2.56	1.30	c.715C>T p.R239X, Nonsense
56	0.18	0.18	38	43	51	13.6	20.96	7.62	16.7	1.41	1.00	c.715C>T p.R239X, Nonsense
57	0	0.18	25	25	6	NA	8.93	3.84	7.49	NA	3.14	c.757C>T p.R253X, Nonsense
58	0.3	0.18	69	70	8	1.7	NA	1.69	4.38	0.02	NA	c.757C>T p.R253X, Nonsense
59	0.6	0.18	41	43	16	1.7	1.51	1.16	0.83	0.42	0.52	c.877C>T p.R293X, Nonsense
60	0.1	0.18	22	23	12	56.15	55.37	36.08	29.94	20.18	25.57	c.715C>T p.R239X, Nonsense

61	0.78	0.48	51	53	23	0.35	0.83	0.25	0.81	0.05	0.01	c.799C>T p.R267X, Nonsense
62	0.18	0.3	44	46	24	3.44	2.59	3.13	2.32	0.16	0.14	c.877C>T p.R293X, Nonsense
63	NA	NA	57	58	18	5.33	12.37	4.14	11.27	0.79	0.73	c.877C>T p.R293X, Nonsense
64	0	0	42	42	11	2.9	3.58	2.53	3.3	0.40	0.31	c.877C>T p.R293X, Nonsense
65	0	0.18	42	43	8	1.66	11.53	1.5	10.16	0.23	1.99	c.130G>T p.G44X, Nonsense
66	0.18	0.18	37	38	20	5.27	11.09	5.19	NA	0.05	NA	c.799C>T p.R267X, Nonsense
67	0.78	1	45	46	12	0.6	0.46	0.54	0.4	0.06	0.06	c.655C>T p.Q219X, Nonsense
68	0.1	0.1	26	27	7	14.71	19.92	14.17	19.61	0.93	0.53	c.808C>T p.R270X, Nonsense
69	0	0	24	24	7	48.17	32.96	39.86	30.05	14.25	4.99	c.808C>T p.R270X, Nonsense
70	-0.08	-0.08	41	43	24	13.32	10.58	10.93	8.88	1.19	0.85	c.940+3delA (intron 7), Splice site
71	0	0.18	43	47	53	4.42	6.02	2.39	3.91	0.46	0.48	c.189+1 G>C (intron 3), Splice site
72b*	2.7	0.48	51	53	24	0.95	1.31	0.56	1.17	0.20	0.07	c.49+3delA (intron 1), Splice site
73*	0	0	27	31	46	8.99	6.03	6.19	3.37	0.74	0.70	c.941-2GT>AG (intron 7), Splice site
74	-0.08	-0.08	27	33	82	45.56	34.96	27.1	19.28	2.71	2.30	c.1245-1G>A (intron 9), Splice site
75	0.18	0.18	23	30	82	21.47	23.43	12.83	15.29	1.27	1.20	c.1245-1G>A (intron 9), Splice site
76	0.18	0.3	21	25	46	62.13	85.43	58.95	67.82	0.83	4.59	c.49+3_49+10delinsGCTT (intron 1) Splice site
77	0.18	0.48	48	51	38	16.69	13.75	14.02	11.42	0.84	0.74	c.116+1G>A (intron 2), Splice site
78	0	0	41	43	25	5.79	6.15	4.07	5.16	0.84	0.48	c.49+2dupT (intron 1), Splice site
79	0.18	0.18	56	59	36	8.42	9.44	8.24	NA	0.06	NA	c.819+1G>T (intron 6), Splice site
80	0.48	NA	63	66	34	0.54	NA	0.39	NA	0.05	NA	c.940+2T>C (intron 7), Splice site

Table S1. Summary of clinical findings and molecular status of 80 CHM patients. * Denotes 7 novel mutations not previously listed in HGMD. [a] REP1 deletion confirmed using western blot analysis. [b] Highlights patients of Asian background. The following sets of patients are siblings (6/7), (28/29), (49/50, (68/69), (74/75).
